# The Ministry of Health

**Published:** 1920-07-24

**Authors:** 


### THE MINISTRY OF HEALTH

The importance of the fact that the State has now assumed the responsibility for the health of its people was hardly in need of further emphasis, but there is little doubt that, from the reading of daily papers, the average person had come to concentrate almost entirely upon the moral and scientific possibilities of the new scheme and to develop a certain degree of impatience at delay, without having due regard to the host of administrative problems with which the Minister and his assistants have been faced. As *The Times* remarks, there has been everywhere a comfortable idea prevalent that the conquest of disease had been advanced, and that in process of time the various scourges which vex our civilisation would now be automatically overcome. Nevertheless, at the moment, although the Ministry has been brought into being, practically the whole of its work has yet to be done. Even its own organisation is incomplete, and still unfinished are the finer developments which will carry its beneficial work to the uttermost corners of the country. It was opportune, therefore, that last week, at the taking of the vote of the Ministry of Health, Dr. Addison should have the chance of giving ample illustrations of the facts as they really are, and we trust that all those interested in health matters have paid due attention to his statement, although the debate which followed it was most disappointing. (See p. 435.)

Housing questions and details of progress in this direction occupied a good deal of his time, and we may on this page be excused for leaving them at the mere reference. The delay, such as all know to have occurred, has a number of definitely circumstantial causes, and we sympathise with the Minister's hope that in obtaining the additional and very necessary labour, a satisfactory scheme could be devised—and the trade unions be brought to agree with it.

On matters more directly medical Dr. Addison's words should be noted and their essential import understood. A sound health policy must inevitably require patience and time for its development, and the first essential is, in any case, the spread of good common information, all of which tends to educate the country towards the principles of prevention of disease. It must be remembered that although much has been done in the publicity sense, yet the Minister himself admitted we have hitherto not made as much real progress as we might have done. Any proper development of a preventive health service must depend upon an adequate supply of trained persons—and there is still in many branches a serious deficiency. This will be quickly remedied, as quickly as circumstances allow, and all endeavour must be made to bring about public appreciation of the value and honourable nature of this type of professional calling. It has, however, been obvious that we cannot expect to produce greatly expanded trained staffs, administrators, doctors, nurses, midwives, inspectors, etc., in one short year. And also in so great a reconstruction as these health problems imply, it is doubtful whether it is not wiser to produce gradually that which must one day come into being. So immense are the problems in hand, so uncertain the outlook in many quarters, that we cannot but feel that there is much to be said both for Dr. Addison's defence against accusations of delay, and for his avowed policy of gradual administrative development.

At least, we know that the existing preventive services in Great Britain, as far as they relate to the immediate surroundings of those individuals they deal with, have reached a stage of perfection commendable in the extreme. It is true that only certain sections of the community came under their care, but they form a very sound basis from which to extend. Also our sanitary services are, internationally compared, remarkably good, and Dr. Addison is undoubtedly right in claiming that the health of the people, many of whom dwell in most pestilential places, is as good as it largely is owing to the activities of the existing official provisions. The foundation of our health services is there, the Ministry represents a new and extending administration, and during its development we must, while appreciating the possibilities, be patient for their complete fulfilment. If we criticise, let it be fairly done.

